# Experimental Elucidation of Templated Crystallization and Secondary Processing of Peptides

**DOI:** 10.3390/pharmaceutics15041288

**Published:** 2023-04-20

**Authors:** Vivek Verma, Isha Bade, Vikram Karde, Jerry Y. Y. Heng

**Affiliations:** 1 Department of Chemical Engineering, Imperial College London, London SW7 2AZ, UK; isha.bade16@imperial.ac.uk (I.B.); v.karde@imperial.ac.uk (V.K.); 2 Institute for Molecular Science and Engineering, Imperial College London, London SW7 2AZ, UK

**Keywords:** pharmaceutical manufacturing, peptides, crystallization, tableting, powder mixing

## Abstract

The crystallization of peptides offers a sustainable and inexpensive alternative to the purification process. In this study, diglycine was crystallised in porous silica, showing the porous templates’ positive yet discriminating effect. The diglycine induction time was reduced by five-fold and three-fold upon crystallising in the presence of silica with pore sizes of 6 nm and 10 nm, respectively. The diglycine induction time had a direct relationship with the silica pore size. The stable form (α-form) of diglycine was crystallised in the presence of porous silica, with the diglycine crystals obtained associated with the silica particles. Further, we studied the mechanical properties of diglycine tablets for their tabletability, compactability, and compressibility. The mechanical properties of the diglycine tablets were similar to those of pure MCC, even with the presence of diglycine crystals in the tablets. The diffusion studies of the tablets using the dialysis membrane presented an extended release of diglycine through the dialysis membrane, confirming that the peptide crystal can be used for oral formulation. Hence, the crystallization of peptides preserved their mechanical and pharmacological properties. More data on different peptides can help us produce oral formulation peptides faster than usual.

## 1. Introduction

Peptides are a category of biomolecules usually with a molecular weight of 500–5000 Da. They closely mimic natural pathways and can exhibit increased potency and high selectivity [1,2]. The development of novel synthesis strategies to produce peptides with modulated pharmacokinetic properties and target-specificity has resulted in more accessible pharmaceutical-grade peptides [3]. Since the synthesis of the first therapeutic peptide, human insulin (a peptide with 51 amino acids), in 1921, more than 80 peptide-based therapeutics have been approved by different drug agencies and have been launched on the market, while more than 500 peptides are in pre-clinical development and 150 in clinical trials [1,4]. As a result of the increased investment and research efforts in the field of peptides, the maturing of peptide synthesis technology, the success of biologics, and pressure on the pharmaceutical industry to maintain approval rates for new drugs, we anticipate peptide therapeutics to continue growing and expanding. Thus, the development of peptide drugs is the most emergent topic in pharmaceutical research.

Regardless of whether a peptide is formed synthetically or expressed using recombinant technology, the purification and/or isolation of peptides are often the bottlenecks of the manufacturing process [5]. High-performance liquid chromatography (HPLC) is the most common technique used for purification and ion exchange. However, the large amount of aqueous/organic waste generated from the purification process makes it an unsustainable process. Alternatively, precipitation can be used to purify and isolate peptides, but precipitation can result in amorphous solids or significant solvent-adsorbed volumes. Therefore, crystallization is often viewed as an environmentally friendly and economically advantageous substitute to chromatographic separations since it uses much less solvent [6]. In addition, crystallization offers us the opportunity to improve particle attributes, such as crystal shape, size, and form. However, the flexible nature of the peptide molecules poses a challenge to their crystallization, therefore requiring researchers to improve the peptide crystallization process [7,8].

The presence of templates (either soft or hard) has been shown to control the crystallization of proteins, such as insulin [9] and lysozymes [10,11], but sometimes it also decelerates the crystallization process by delaying the induction time [12]. Link and Heng have demonstrated the influence of dissolved amino acids acting as soft templates for insulin crystallization. The intermolecular interactions between insulin and the l-arginine molecule in their study led to insulin stabilization, resulting in an improvement in the crystal occurrence compared to that of l-glycine [9]. Similarly, Li et al. confirmed that the presence of silica particles facilitated the crystallization of lysozymes with an improved induction time and crystal yield [11]. Furthermore, the presence of glass beads accelerated the nucleation of glycine, diglycine, and triglycine. This was potentially due to the enhanced number of probable non-covalent interactions between the hydrogen bond donor of glycine and its homopeptides and the hydrogen bond acceptor of the glass beads. This was further corroborated with the molecular dynamics simulations, which confirmed the enhanced residence time of triglycine on the surface of the glass beads due to an increased number of hydrogen bond interactions [13,14].

Although crystallization could potentially improve the purification and isolation of peptides, the use of peptide crystals in drug products is far from a reality. In addition to the advantage of target specificity and selectivity, the unfavourable physicochemical properties of peptides, such as large molecular weight, inactivation by gastric pH values, hydrophilicity, low intestinal permeability, and susceptibility to digestive enzymes, work against successful oral peptide delivery [15]. Therefore, the most widely used route of peptide administration is the parenteral route, which is generally inconvenient, painful, and requires medical knowledge. Although, many drugs other than peptides, such as proteins, monoclonal antibodies, small molecule drugs, etc., are administered parenterally, it is estimated that ~5% of the population strongly prefers other routes of drug administration [16]. Therefore, the oral delivery route is considered as the preferred route of administration as it is non-invasive and has a high level of patient compliance. Additionally, it provides a chance to extend the patent life of expiring injectable peptides with novel formulations.

The oral delivery of peptides is influenced by food and water intake [17]. Further, the inherent physical and chemical properties of peptides, such as molecular size, proteolytic stability, hydrophilicity, and ionic charge, also influence their oral absorption [18]. Although oral peptides and proteins face barriers to delivery, the number of formulations that have been launched or are in clinical trials is steadily increasing. There are multiple strategies for improving the oral delivery of peptides, including permeation enhancers (PEs), multi-particle systems, targeted particles, nanotechnology, enzyme inhibitors, colonic delivery methods, and modifications to the peptides themselves (cyclisation or the use of non-natural amino acids) [19,20,21,22,23]. Since the publication of the first paper reporting the use of alcohol to improve the oral absorption of insulin in 1923 [24], other researchers have also attempted to develop peptide oral formulations. This led to the approval of Sandimmune^®^, the first oral formulation of a cyclic peptide, cyclosporin A, by the FDA in 1990, followed by the approval of Neoral^®^ developed by Novartis, which was an updated formulation of Sandimmune^®^ [22]. Later, oral semaglutide (Rybelsus^®^ in 2019) and oral octreotide (Mycapassa^®^ in 2020) were approved by the FDA. These formulations demonstrated that the oral delivery of peptides is feasible if the peptides and formulations are optimized for the routes of administration. Crystallization offers peptide stability, therefore diversifying the potential formulations for various routes of administration, including oral dosage. Additionally, it offers the controlled release of the peptide from the polymeric encapsulation, leading to an improvement in the half-life of peptides [25]. Peptide crystals further allow for high-concentration doses without increasing the viscosity of the suspension as well as the ability to reach a concentration greater than 200 mg/mL in the final dosage form, leading to a higher bioavailability and lower dosage requirement [25]. Hence, peptide crystallization offers great applicability for oral doses of the current peptide molecules on the market.

At present, oral formulations are available for several hormones, including insulin, vasopressin, somatostatin, calcitonin, parathyroid hormone (PTH), uroguanylin, thyroid hormone-releasing hormone, and GLP1. These treatments can be classified into two categories based on their intended action within the body—those that require oral absorption and those that require retention in the gastrointestinal tract [26,27]. All of these formulations use one of the above-mentioned strategies; more specifically, well-known permeation enhancers such as sodium caprate (C10; also known as decanoic acid) and sodium N-[8-(2-hydroxybenzoyl)amino] caprylate (SNAC; also known as salcaprozate sodium) improve the transcellular and paracellular permeation [20]. However, there has not yet been a report of an oral formulation of the peptide using just the peptide crystals on their own. This is potentially due to the fact that no peptide drug is small enough to be compliant with Lipinski’s rule of five (MW < 500 g/mol, H-bond donors < 5 and H-bond acceptors < 10, LogP < 5, rotatable bonds < 10, and total polar surface area < 140 Å^2^) for predicting good absorption and permeation [28]. Surprisingly, the peptides that have been identified as having good oral activity and that could be potential candidates for oral formulations, in addition to the one in the clinical trial, are mostly cyclic [29]. This suggests that further research is needed to explore more cyclic peptides with good oral activity.

This work focuses on improving the crystallization of peptides using the templated crystallization approach, already established by the authors [9,10,13,14]. The templates interact with the crystallising solute through functional group complementarity, allowing the sequestration of solute molecules on the surface of the templates with h-bond interactions for a long enough time to achieve a fully grown crystal. This was recently complemented with a molecular dynamics simulation, as mentioned earlier in the section [14]. In this work, diglycine was crystallised in the presence of different pore sizes of silica particles to examine the effect of template pore size on the crystallization time and rate. Diglycine is made by combining the simplest amino acid, i.e., glycine, with itself though a peptide bond, making it the smallest known peptide, which behaves similarly to a small molecule due to the lack of degrees of freedom, defined unit cell, and well-defined intermolecular contacts. The crystallised peptide was later blended with a widely used pharmaceutical excipient, microcrystalline cellulose (MCC) [30], to obtain an oral formulation. The formulation was later compressed to form tablets, which were studied to obtain the tabletability, compressibility, and compactibility for the peptide tablets. This is the first study reporting the tablet properties of a peptide formulation. The tablets were later studied to obtain the permeability rate of different formulations.

## 2. Materials and Methods

### 2.1. Materials

Diglycine (Digly, >99% by titration) and microcrystalline cellulose (MCC, Avicel^®^ PH-101, 50 µm particle size) were supplied by Sigma-Aldrich and used as received. Deionized (DI) water was supplied by a Sartorius Arium^®^ Advance (Göttingen, Germany). Silica particles (40–63 µm) of different pore sizes (6 nm, 10 nm, 30 nm, and 50 nm) were purchased from Element and used as received.

### 2.2. Isothermal Colling Crystallization Experiments

Mettler-Toledo EasyMax 102 was used for the cooling crystallization experiments, allowing for precise control over experimental variables, such as reactor temperature, stirring rate, and heating and cooling rates. A diglycine solution (concentration of 284.28 mg/mL, total volume of 40 mL, and saturation temperature of 40 °C) was prepared in DI water, as per the diglycine solubility data published previously by our group [31]. The saturated diglycine solution was heated to 5 °C above the saturation temperature for complete dissolution of diglycine. This solution was cooled to 32.7 °C to induce crystallization at relative supersaturations of 1.20, which is in the metastable zone width limit, enabling us to capture the effect of templates on heterogenous nucleation of diglycine. The change in concentration upon nucleation of diglycine in the absence and presence of porous silica particles ((10% *w*/*w* loading) was captured using Mettler-Toledo ReactIR 15 system, an in situ Fourier transform infrared (FTIR) probe. Each experiment was carried out at least twice to ensure the reproducibility of the results. The induction time was accessed from the desupersaturation curves using the tangent method that was previously used by the authors [14].

### 2.3. Dynamic Light Scattering (DLS)

DLS was carried out to measure the hydrodynamic radii of diglycine in water. A dilute solution of glycine was prepared, filtered using a disposable syringe filter with a pore size of 0.2 µm, and analysed using Zetasizer μV (Malvern, UK). Samples with a polydispersity index lower than 0.10 were used for the size measurements.

### 2.4. Solid State Characterization of Isolated Solids

#### 2.4.1. Powder X-ray Diffraction (PXRD)

PXRD was recorded for the diglycine porous silica composites isolated after the isothermal cooling crystallization experiments using a PANalytical Empyrean diffractometer (Malvern Panalytical, Malvern, UK) with a Cu radiation source (λ = 1.541 nm) at 40 mA and 30 kV. Scans were performed between 5 and 35° 2θ at a scan rate of 0.013° 2θ/min.

#### 2.4.2. Scanning Electron Microscopy (SEM)

The as-received and isolated samples from the crystallization experiments were analysed for the surface morphology and crystal habit using SEM (Zeiss LEO Gemini 1525, Cambridge, UK) at a working distance (WD) of 5–8 mm and a voltage of 5 kV.

#### 2.4.3. True Density Measurement

The Micromeritics^®^ Accupyc II helium gas displacement pycnometer was used to measure true density of the samples at ambient temperature (25 °C) based on USP 699 standard procedure. True density measurements were perfomed using the 1 cm^3^ sample cell and 20 purge cycles.

### 2.5. Tabletting of the Diglycine–Silica–MCC Composite

The diglycine–silica–MCC composite tablets were compressed using a Gamlen R-series compaction simulator (Gamlen Tableting Limited, Beckenham, UK). Composite tablets of 100 mg (22.5 mg diglycine, 2.5 mg silica, and 75 mg MCC) were prepared using a cylindrical 6 mm punch die at a punch speed of 10 mm/min. All the samples were compacted at compaction pressures of 1–3 kN with a step increase of 0.5 kN to thoroughly study the compaction behaviour of diglycine–silica–MCC composite powder. MCC was also compressed at the same compaction pressure as the composite and acted as the positive control. The tensile strength of the tablets was measured using the EZ50 equipment (Lloyd Instruments, Bognor Regis, UK) equipped with a load cell on 10 N and an operating speed of 0.1 mm/min. The tabletability, compactability, and compressibility of the powder were evaluated to assess its mechanical properties.

#### 2.5.1. Tablet Porosity

Equation (1) was used to calculate tablet porosity.

(1) $$Tablet\;porosity=1-\frac{\rho_{app}}{\rho_{true}}$$

The *ρ_true_* is the true density (g/cm^3^) calculated using the helium pycnometer mentioned in Section 2.4.3, while *ρ_app_* is the apparent density calculated by dividing tablet weight by its volume.

#### 2.5.2. Tabletability

The tabletability of a material is represented by the relationship between its tensile strength and the compaction pressure applied. Tabletability is expressed by a linear relation between tensile strength and compaction force, as given by Newton et al. [32] and presented in Equation (2).

(2) $$\sigma_{t}=C_{p}P+b$$

where *P* is the compaction pressure, *C_p_* is the tabletability parameter, and *b* is a constant.

#### 2.5.3. Compactability

An exponential relation between the tensile strength and porosity expresses the compactability of a composite. Ryshkewitch–Duckworth proposed a mathematical equation to understand compactability, as shown in Equation (3) [33].

(3) $$\sigma_{t}=\sigma_{t0}e^{\left(-bP\right)}$$

where tablet tensile strength (MPa) is represented by *σ_t_* and tablet tensile strength at zero porosity (MPa) by *σ_t_*_0_. The tablet porosity is given by *P*, while *b* is an empirical constant representing bonding capacity, in which stronger bonding between primary particles is expressed by higher *b* value [34].

#### 2.5.4. Compressibility

Compressibility profile shows the change in the tablet porosity with the increasing compaction pressure. A tablet with low porosity is associated with the capping problem and can result in slow tablet dissolution. Compressibility can be assessed by change in tablet porosity with compaction pressure as expressed by Heckle’s model according to Equation (4).

(4) $$-\ln\varepsilon =\ln\left(\frac{1}{1-D}\right)=kP+A$$

The compressibility of a powder can be indicated by the values of the Heckle coefficient (*k*) and its reciprocal giving yield pressure (*P_y_*). In the Heckle’s model, tablet density, relative tablet density, compression pressure, and intercept are represented by *ε*, *D*, *P*, and *A*, respectively.

### 2.6. In Vitro Diffusion Studies

The diffusion profile of diglycine forming the composite tablets was studied using a dialysis tube as a substitute for the intestinal membrane [35]. Diffusion tests were completed using the Pur-A-Lyzer™ Maxi Dialysis Kit-Maxi 6000 (Sigma-Aldrich Co., LLC, London, UK) with a molecular weight cut-off of 6–8 kDa and volume capacity of 0.1–3 mL. The diffusion was performed in 100 mL water in a Duran flask, at 37 °C in a water bath, and stirred at 100 rpm. The dialysis tubes were soaked in the diffusion media for 30 min before adding the tablet. The dialysis tube contained 2 mL water and the composite tablet, while the flask contained 98 mL water, making a total volume of 100 mL. All the diffusion experiments were repeated at least three times on different days to exclude human error. Samples of 1 mL aliquots were withdrawn from the diffusion flask at fixed times of 1, 3, 5, 10, 15, 30, 45, 60, 90, 120, and 1440 min, and were analysed by UV spectrophotometry at 215 nm. A calibration curve was previously made at this wavelength with an R2 value of 0.99 and was used for the determination of concentration.

## 3. Results and Discussion

**Figure 1:**
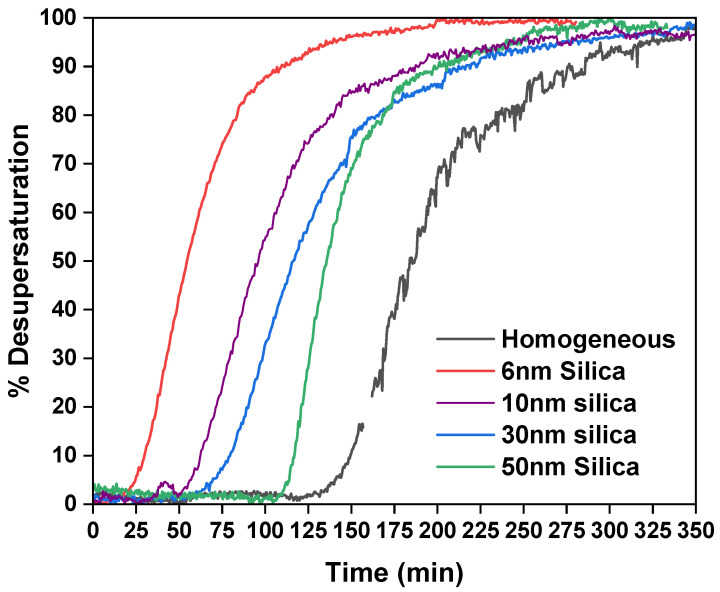
Comparison of % desupersaturation curves of diglycine in the absence and presence of porous silica at S = 1.20; volume = 40 mL; T_sat_ = 40 °C; T_cry_ = 32.7 °C.

The induction time of the diglycine crystallised in the absence and presence of the porous silica templates is presented in Figure 1. The silica templates have a positive yet discriminating effect in that they reduce the time required for crystallising diglycine at a supersaturation of 1.20. The significant reduction in the induction time (the time required to observe a significant change in the solution concentration from the isothermal holding time at the crystallization temperature) [30] of diglycine was observed when it was crystallised in the presence of silica with a pore size of 6 nm.

The reduction in the induction time in the presence of porous silica has a direct relation with the pore size. The smallest silica with a pore size of 6 nm exhibited a five-fold reduction in the induction time compared to that of the homogeneous nucleation, as presented in Table 1. This was followed by silica with pore sizes of 10 nm, 30 nm, and 50 nm, which had 3-fold, 2-fold, and 1.5-fold reductions, respectively.

This direct relation of pore size to the reduction in the induction time is potentially due to the diglycine cluster size matching of pore sizes. DLS data suggest a hydrodynamic radius of 0.94 nm for diglycine in water, while a unit cell of diglycine contains four diglycine molecules [13], bringing the diglycine cluster size in the range of silica with a 6 nm pore size. A pore size (length) of 6 nm is sufficient to sequester a critical size cluster of diglycine, creating local supersaturation and resulting in the nucleation of diglycine in the pores. Similar results were obtained by Shah et al. [36] when different-molecular-weight proteins were crystallised using the templates with engineering pores with an optimum size similar to their hydrodynamic radius. Further, an antibody named anti-CD20 was crystallised using the specifically designed porous silica template with a pore size in the range of the molecular diameter of the antibody [37,38]. Silica with a 10 nm pore size also influenced the diglycine induction time; 6 nm and 10 nm pores are significantly not different, and hence are also able to crystallise diglycine and reduce the induction time. Due to the large pore size of the other pores’ silica, the diglycine induction time was not affected much by the other pores’ silica compared to the silica with a pore size of 6 nm.

**Figure 2:**
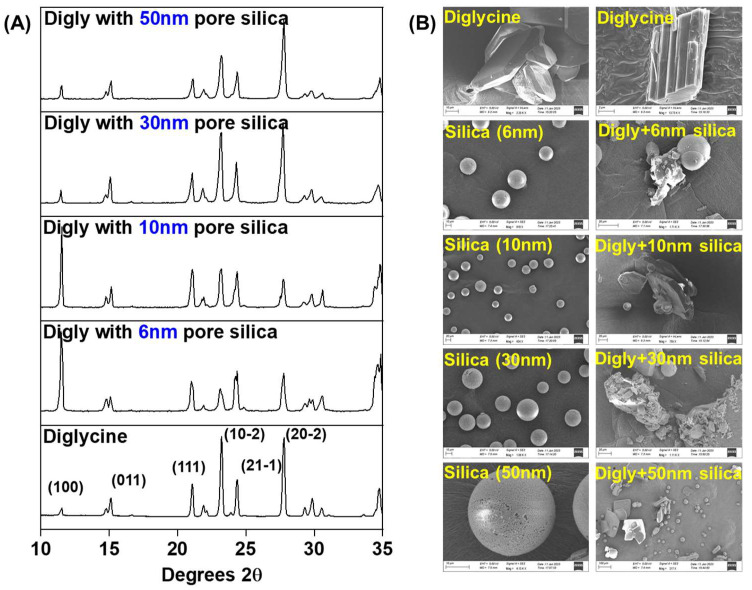
(**A**) Powder X-ray diffraction spectra of the diglycine–silica composite solids isolated upon complete desupersaturation of diglycine in the presence of porous silica at S = 1.20, along with the diglycine patterns; (**B**) Scanning electron microscopy images of diglycine, porous silica, and isolated solids after the desupersaturation experiments in the presence of porous silica.

Figure 2 presents a solid-state analysis of the isolated composite solids from the desupersaturation experiments in the presence of different-pore-size silicas. The PXRD graphs in Figure 2A show the crystallization of diglycine stable form (α-form) in the presence of porous silica. There is a preferred orientation observed for the peaks at (100) and (20–2) when crystallised in the presence of porous silica. This is potentially due to the non-interaction between the functional groups (-COO, carboxyl group) present on these surfaces with the silica hydroxyls. Figure 2B presents the SEM micrographs of the isolated solids along with the bare silica and diglycine. The diglycine crystals obtained in the presence of porous silica seem to be associated with the silica particles, as observed in the SEM micrographs, suggesting the crystallization of diglycine either on the silica surface or the pores. The particle size of the diglycine crystals obtained is in the range of 20–100 µm.

**Figure 3:**
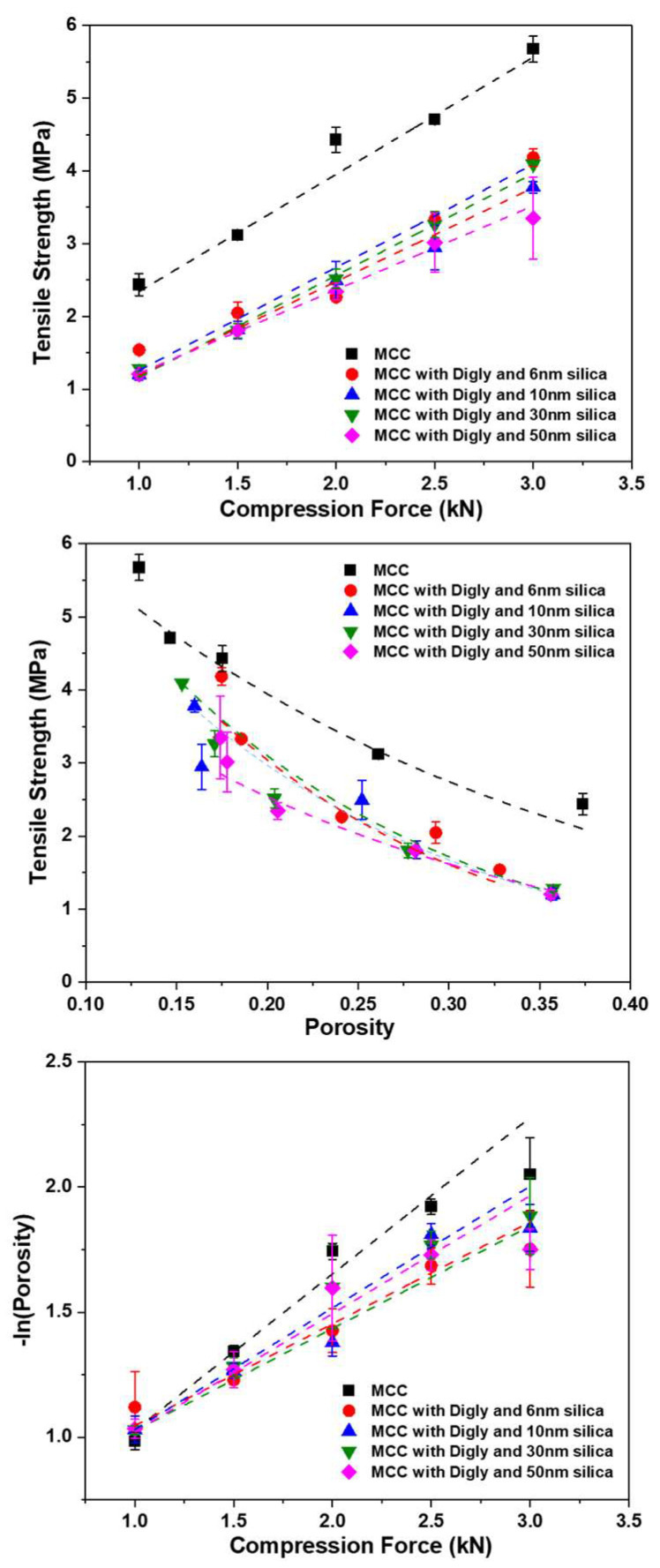
(**Top**) Tabletability, (**Middle**) compactability, and (**Bottom**) compressibility profiles of the 25% loading blend of diglycine–silica–MCC composite tablets prepared along with MCC alone (blue squares, PH101). Red circles, blue upwards triangles, green downwards triangles, and pink rhombuses represent diglycine crystallised using silica with pore sizes of 6 nm, 10 nm, 30 nm, and 50 nm as templates, respectively, with n ≥ 3 (n is the number of experiments).

The physicochemical and pharmacodynamic behaviours of pharmaceutical tablets are influenced by their mechanical strength [39,40]. A comparison of the tabletability, compressibility, and compactability profiles of the diglycine–silica–MCC composite tablets was conducted. This is the first study reporting the mechanical properties of the simplest peptide of glycine, diglycine. Tabletability is a function of tensile strength and compaction force. The tabletability profile of the diglycine–silica–MCC composite along with the pure MCC is presented in Figure 3 (top). As a widely used pharmaceutical excipient, MCC has been extensively studied and reported in the literature, and can be easily compressed with an excellent tensile strength. Hence, MCC was selected as the excipient to be used in this study [41]. Due to its easy compressibility, the tensile strength of MCC is >2 MPa at any compression force, while the tensile strength of the diglycine composite is >2 MPa after 2 kN, irrespective of the silica pore size. Pharmaceutical tablets exhibiting a tensile strength greater than 2 MPa are considered as passing the standard requirements for manufacturability, quality, and biopharmaceutical performance [42]. The tabletability parameter (*C_p_*) of the tablets can be obtained using the Newton equation [32]. The *C_p_* value of 1.61 exhibited by MCC is the highest (reported in Table 2) compared to that of the diglycine composite, indicating tablets for the diglycine composite are less stable compared to MCC. This is due to the presence of glycine crystals in the composite tablets.

Figure 3 (middle) presents a tensile strength vs. porosity graph, representing the compactability of the tablets. Compactability is the ability of a powder to reduce porosity under an applied pressure. The curve-fitting parameters obtained (reported in Table 2) using the Ryshkewitch–Duckworth equation [43] were used to analyse the compactability of the composite tablets. The tensile strength at zero porosity (*σ_t_*_0_) is higher for all the composite samples except for the silica composite with a 50 nm pore size compared to that of MCC. This suggests that the porosity of the composite tablets is less than that of MCC. The composite with silica with a 50 nm pore size could potentially have more agglomerated samples, resulting in a high porosity in the tablets. Further, a higher value of the constant ‘*b*’ represents a stronger bonding between the primary particles [34]. The value of ‘*b*’ is higher for all the composite samples compared to that of MCC, suggesting that the interparticle bonding is higher in the composite tablets than the MCC tablets, which is due to the presence of silica in the composite tablets filling the interparticle distance, as also observed in the SEM micrographs (Figure 2B) with the silica particles associated with the diglycine crystals.

The compressibility of the powders was assessed using the Heckle equation. The values for the Heckle coefficient, *k*, which were obtained from the slope of the Heckle plot, and its inverse values, i.e., the yield pressure, (*P_y_*), for the different composite systems along with that of pure MCC are presented in Table 2. The high value of *k* and the low value of *P_y_* suggests the good plasticity of MCC. On the contrary, the diglycine composite exhibit an inverse behaviour to that of pure MCC, with low values for *k* and higher values for *P_y_*, indicating poor plasticity and low compressibility properties. This is due to the presence of diglycine crystals, which exhibit poor compressibility, leading to high porosity in the tablets.

There were no differences observed in the flow properties of the formulations, and this potentially was due to the presence of 75% *w*/*w* microcrystalline cellulose (MCC) in the formulation mixture. This made the formulation mixture flow more like pure MCC. The silica tested in the study were mostly identical from outside in terms of their shape, size, and functionality, while the only difference was in their internal pore diameter. Due to the identical nature of the silica tested, there was no obvious difference in the flow properties. Moreover, variations in the particle size distribution of the crystallised diglycine will also influence the flow properties of the formulation mixture. Particles with a larger crystal size distribution are more likely to have poor flow properties due to the plate-shaped diglycine crystals resisting a smooth flow, whereas this may not be the problem with crystals of a smaller size distribution. However, this was not observed with the crystals in this work, as all the crystalline particles obtained from the templated crystallization had a similar size distribution, as can be seen in the SEM micrographs in Figure 2B. A similar phenomenon was observed by a group member in the past, confirming that elongated needles were more cohesive than hexagonal crystals, which is due to the combined effects of surface energy and surface area leading to poor flow behaviour due to the high cohesivity of the crystals [44].

The diglycine–silica–MCC tablets were subjected to the diffusion test using the dialysis tube to observe their diffusion rate from the dialysis membrane. Diglycine is readily dissolved in water based on its solubility data, which have already been published in the literature [31], and hence, performing the dissolution study is futile. In contrast, the low intrinsic permeability of peptides is a bottleneck in the oral delivery of peptides. The oral absorption of peptides in the body occurs through three possible pathways: [18]

- paracellular, involving passing in between enterocyte cells, only allowing smaller peptides with a low molecular weight;
- transcellular, involving passage through the enterocyte cell with the help of other cells;
- ligand-assisted transport, allowing transportation through the intestinal mucosal membrane using a permeation enhancer.

The molecular weight, flexibility, and hydrophilic nature of smaller peptide-based drugs provide them the advantage of paracellular passage in between cells [18]. This phenomenon was later confirmed by Foger et al. [45] in a report that illustrated that the peptide permeability increases as the molecular weight decreases, but only for peptides with a molecular weight up to 1.4 kDa.

**Figure 4:**
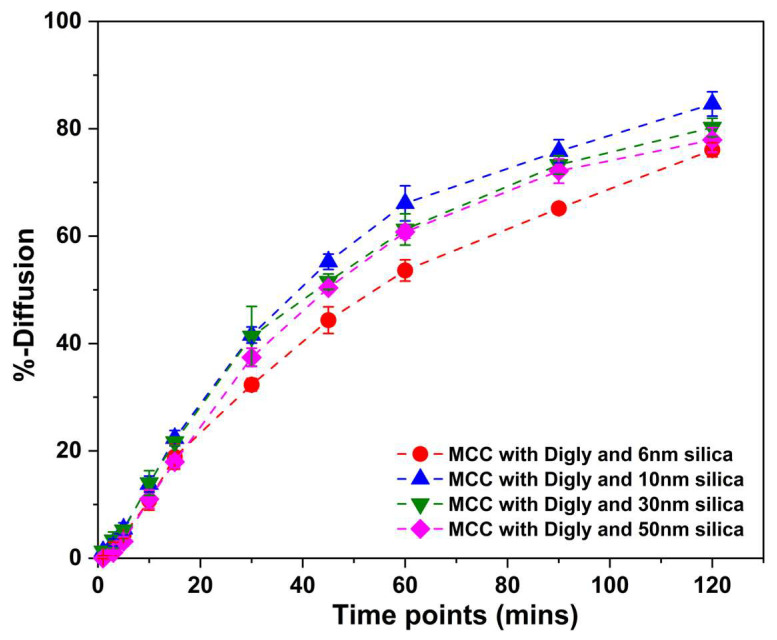
%diffusion of diglycine from the diglycine, silica, and MCC composite tablet compressed at 2.5 kN. The diffusion medium was water; volume = 100 mL; and stirring = 100 rpm.

Figure 4 presents the diffusion of diglycine over a period through the permeation membrane with a molecular weight cut-off of 6–8 kDa. The diffusion rate experiments were performed using the diglycine composite tablets compressed at 2.5 kN, as the tablets had the desired tensile strength of more than 2 MPa at this compression force. All the tablets exhibited an extended-release profile, confirming their easy diffusion through the permeation membrane with approximately a 50% diffusion achieved in the first hour and a complete dissolution in 12–16 h. This steady diffusion suggests that permeation enhancers are not required for small peptides, as mentioned by Klepach et al. [18].

Although diglycine was crystallised in the presence of different-pore-size silica, except for influencing the induction time of diglycine, the porous silica had a minimal impact on the mechanical properties of the tablets. The presence of silica improved the tablet properties by reducing the tablet porosity, thereby increasing the tensile strength of the tablets. Therefore, templated crystallization using pharmaceutical excipients as the templates could potentially not only improve the downstream processing time, but also improve the particle attributes, such as crystal size, shape, and form, also improving the mechanical properties of the tablets. However, more work is needed in this area to prove the ability of templates to crystallise larger peptides and formulate them in oral tablets to study their mechanical properties.

## 4. Conclusions

In this study, diglycine, the simplest glycine homopeptide, was crystallised in the presence of porous silica, presenting a template-assisted crystallization of peptides. The induction time of diglycine was significantly reduced in the presence of silica with 6 nm and 10 nm pore sizes due to the size complementarity in the cluster size of diglycine and the pores. The induction time of diglycine had a direct relationship with the silica pore size. The induction time increased with the increase in the silica pore size, but the presence of silica had a positive and discriminating effect. Further, our PXRD graphs suggested the crystallization of a stable α-form in the presence of porous silica, while all the crystals were seen to be associated with the silica particles in our SEM micrographs. The mechanical properties of the diglycine–silica–MCC tablets were accessed through tabletability, compactability, and compressibility curves. The mechanical properties of the tablets remained uninfluenced by the presence of diglycine–silica and presented similar tablet properties as those of MCC alone. Lastly, our diffusion analysis of the diglycine composite tablets presented an extended release with a constant flux of diglycine through the membrane. The oral formulation of peptides is not limited to diglycine, and hence more research is needed in the oral delivery of peptides.

## Figures and Tables

**Table 1:** Average induction time of diglycine in the absence and presence of porous silica at S = 1.20; volume = 40 mL; T_sat_ = 40 °C; T_cry_ = 32.7 °C.

Pore Diameter	Induction Time (min)
Homogeneous	145 ± 7
6 nm pore	29 ± 5
10 nm pore	43 ± 17
30 nm pore	72 ± 3
50 nm pore	115 ± 7

**Table 2:** Summary of the mechanical parameters obtained from the curve fitting of tabletability, compressibility, and compactability curves for diglycine–silica–MCC composites and bare MCC tablets (*C_p_* is the tabletability parameter, *σ_t_*_0_ is the tablet tensile strength at zero porosity, *b* is an empirical constant representing bonding capacity, *k* is the Heckle Coefficient, and *P_y_* is the yield pressure).

Sample	Tabletability	Compactability		Compressibility	
	*C_p_*	*σ_t_*_0_	*−b*	*k*	*P_y_* (kN)
MCC	1.61	7.98	3.30	0.54	1.84
Digly-6 nm pore size Silica-MCC	1.33	10.40	5.82	0.34	2.90
Digly-10 nm pore size Silica-MCC	1.26	7.95	5.16	0.43	2.32
Digly-30 nm pore size Silica-MCC	1.41	8.34	5.38	0.44	2.27
Digly-50 nm pore size Silica-MCC	1.10	7.60	5.19	0.38	2.64

## Data Availability

Not applicable.
